# Maxillary Midline Diastemas in West African Smiles

**DOI:** 10.1016/j.identj.2022.06.020

**Published:** 2022-07-31

**Authors:** Sefaakor Ahiaku, Brian J. Millar

**Affiliations:** Faculty of Dentistry, Oral & Craniofacial Sciences, King's College London, London, United Kigdom

**Keywords:** Perceptions, Maxillary midline diastema, West Africa, Gap tooth, Attractiveness, Desirability

## Abstract

The maxillary midline diastema (MMD) has experienced a renaissance in popular culture over the last 10 years. Current literature states that MMDs should be eliminated where possible or be reduced to 1 mm or less in width. This literature review explores the evidence for this in a West African population. A systematic search of the literature (Medline and Google Scholar) followed by a hand search was carried out to identify all papers evaluating perceptions of MMDs. In all, 209 papers were identified in the literature search, 8 of which met the inclusion criteria. The review revealed that midline diastemas are desirable in this specific population with a width of 2 to 4 mm deemed as attractive. As many as 95% of participants found the MMD beautiful, and as many as 95% would consider having one created artificially. The literature does not support the position that MMDs larger than 1 mm should always be treated to be perceived as attractive in this population. MMDs are accepted as attractive and should be considered a key feature in smile design for this population.

## Introduction

### Definition

**Fig. 1:**
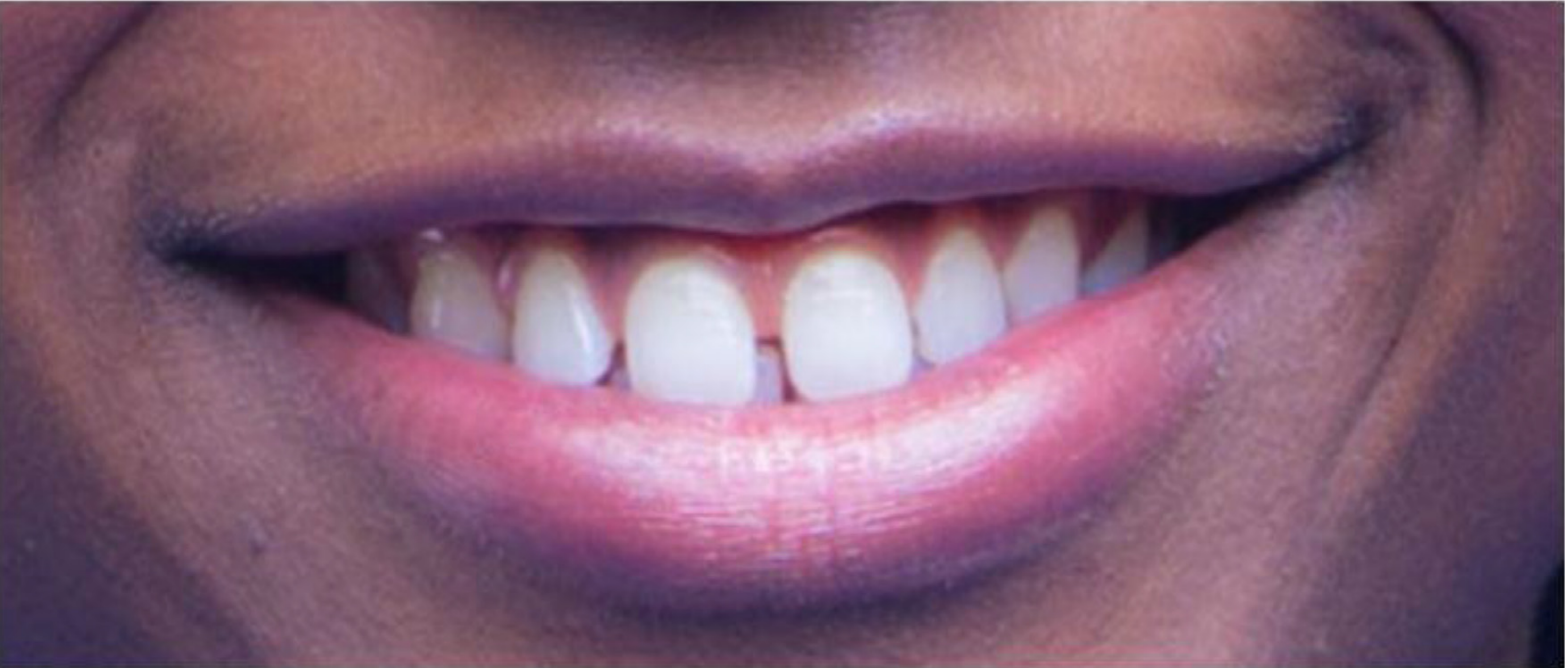
Maxillary midline diastema.

A maxillary midline diastema (MMD) is a space between the 2 central incisors irrespective of its size, and it is considered a normal and naturally occurring phenomenon (Figure 1). In some instances, the absence of contact between the teeth may result in a “black space” which some may perceive to be unaesthetic.

### Causes

MMDs occur for many reasons and may be developmental or acquired (Table 1). McVay and Latta^1^ found the overall incidence of a midline diastema to be 22.33% but found the incidence overall to be higher in Black populations, who also had a higher rate of wider diastemas. The developmental causes have been noted to affect as many as 36% of people in a Black population^2^ and as few as 3.5% in a Caucasian population.^3^ The group most commonly impacted are those aged between 12 and 18; some diastemas disappear during this period without intervention.^4^ In the West, this period often coincides with a referral for orthodontic treatment to minimise the perceived aesthetic detriment of these spaces to an aesthetic smile.

**Table 1:** Causes of midline diastemas.^4^^,^^5^

Developmental causes	Acquired causes
Impacted teeth in the anterior maxilla (mesiodens)	Periodontal disease, causing labial proclination and/or drift of teeth
Supernumerary teeth	Open bites associated with habits like thumb-sucking or a protrusive tongue thrust
Muscle attachment high on the ridge (labial frenum)	Primary or secondary occlusal trauma
Small teeth (hypodontia) +/- peg-laterals or absent laterals	Drift and/or proclination of teeth caused by a poorly designed occlusion
Proclined arrangement of incisors	Trauma changing tooth position
Tooth size/arch shape discrepancy	Artificial creation
Impacted canines	
Rotated incisors	

### Perceptions

Whilst perceptions about midline diastemas are multifactorial and complex, racial and national variation in these perceptions have been noted.^6^ It is a commonly held premise in Western populations that MMDs are unaesthetic and should be eliminated where possible or at least reduced to 1 mm or smaller.^7^ There are, however, notable exceptions to this in the media.^8^^,^^9^ This perception is underpinned by the belief that anything larger than 1 mm would be distracting to the eye.^10^ In addition, symmetry and parallelism across the midline have been noted as highly desirable, with smiles that exhibit this—alongside tooth dimensions that reflect the golden proportion—being deemed as the most attractive.^11^ These aesthetic ideals are commonly taught in dental schools and found in textbooks and in papers. There is a distinct lack of literature outside the Western world and the Middle East in this area (Table 4).

In West Africa, perceptions about midline diastemas and spacing in general differ significantly from those often expressed in the literature. A “gap” is often considered a sign of beauty^4^ and an indication about future wealth and luck amongst some groups. As a result, individuals are less concerned about the presence of a diastema, with larger diastemas of 2 to 4 mm being more commonly accepted.^5^ Newman-Nartey et al^4^ found in their study that amongst patients who had diastemas ranging from 0.56 to 10.48 mm, the chief complaint was most frequently proclination of the central incisors, followed by generalised spacing, as opposed to the presence of the diastema itself.

### The dentist's role

As dentists, it is common for us to provide treatment in the aesthetic zone that results in a dramatic change in a patient's smile. Black triangles and other spaces in the smile are frequently seen as problematic for patients and dentists alike^46^; therefore, treatments aimed eliminating these features in the smile have become particularly popular with the advent of cosmetic composite bonding, porcelain veneers, and adult orthodontics. These treatments have become readily available to the wider population, and in the last 15 years, there has been an increase in patients wanting this type of care. Many patients are willing to travel abroad to have these treatments at a lower price (in the case of those based in the West) or to access them in general (for those based in West Africa).^47^

When planning a smile design case, it is important that we consider cultural and racial factors as they pertain to our patients. This understanding could help inform our treatment plans and ensure that our patients are making informed decisions. Whilst for some patients, having aesthetic dental treatments is a status symbol, it is important that we recognise that treatment options are patient-specific. Providing a smile that is culturally and racially unusual may cause an imbalance.

## Methods

A literature review was carried out to answer following questions:

1. Are MMDs desirable/aesthetic in a Black West African population?
2. Should MMD design be considered as an integral part of smile design in a Black West African patient?
3. How can the quality of the evidence in this region be improved?

A literature search was conducted using the Medline database to search for papers that looked at perceptions about diastemas using keywords and subject headings (Table 2). This search returned 114 papers.

**Table 2:** Key words and subject headings used in literature search.

Key words	Subject headings
Diastema	Diastema
Perception	Perception
Smile aesthetics	Visual perception

Hand-searching was then conducted by looking at reference lists of these papers and conducting a Google Scholar search. Screening reference lists yielded a further 82 papers that were not on the original list, and Google Scholar yielded a further 12.

The total number of papers identified was 208. The titles and abstracts of these papers were screened for relevance. Those papers that passed screening were subsequently retrieved, read, and scored against the inclusion criteria for eligibility (Table 3). One paper was inaccessible.^48^ One paper from the literature search and 7 from hand-searching were deemed to fit the inclusion criteria.

**Table 3:** Inclusion and exclusion criteria for the literature review.

Inclusion criteria	Exclusion criteria
Based in a West African population	Sample only includes participants who are dentally influenced
Ascertains perceptions	Paper published before 1990
Adult participants in the sample (16+ years)	Fewer than 50 participants in the observed sample
Where photos are included in the study, the photos are of are adults (16+)	Paper not peer-reviewed
Statistical analysis carried out on data set	
Evidence levels 1–3	
Demographic information on participants documented	

**Fig. 2:**
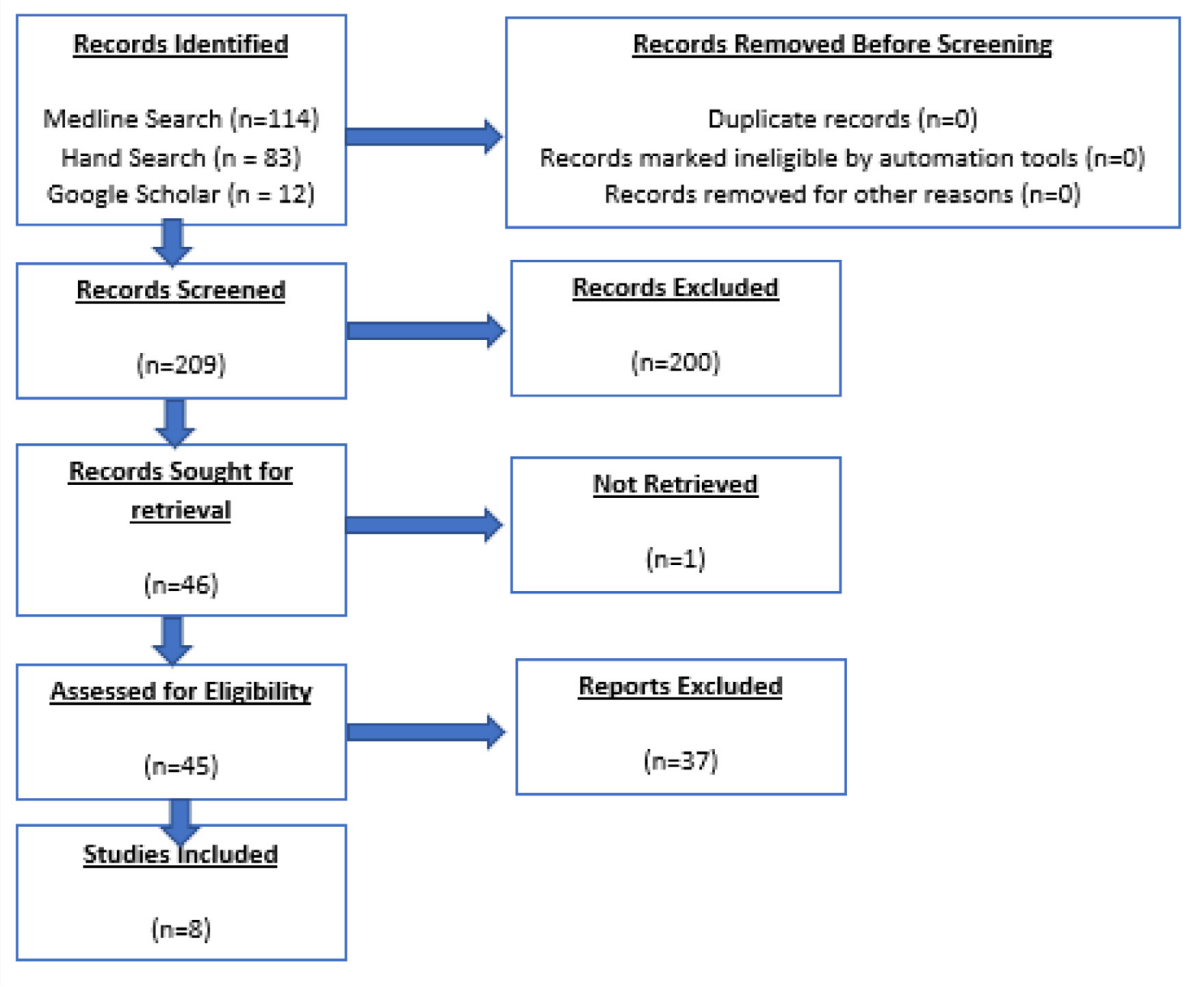
PRISMA flow diagram.

The final number of papers included in the literature review was 8^2^^,^^5^^,^^49^, ^50^, ^51^, ^52^, ^53^^,^^54^ (Figure 2). To understand this data in a global context, a summary of the 37 papers that met the criteria but did not look at a West African population are also included in Table 4.

**Table 4:** Summary of worldwide literature on maxillary midline diastemas.

Region	Paper	Location	Participants	Findings	Ideal diastema width
**Middle East**	Abu Alhaija et al, 2011^12^	Jordan	600		≤1 mm
	Aldeeri et al, 2020^13^	Saudi Arabia	216		≤1 mm
	Al-Rubayee et al, 2017^14^	Iraq	200	46.4% of those with a midline diastema accepted it as desirable	
	Alhammadi et al, 2018^15^	Saudi Arabia	408		0 mm
	Al Taki et al, 2016^16^	Dubai	110		≤0.5 mm
	Geevarghese et al, 2019^17^	Saudi Arabia	200		0 mm
	Kadhom & Sadoon, 2019^18^	Iraq	600		0 mm
	Mokhtar et al, 2015^19^	Saudi Arabia	260		0 mm
	Nazeh et al, 2016^20^	Jordan	200		≤1 mm
	Talic et al, 2013^21^	Saudi Arabia	60		0 mm
	Ousehal et al, 2016^22^	Saudi Arabia	60		≤2 mm
**Europe**	Aramalaite et al, 2018^23^	Lithuania	431		0 mm
	Aljefri & Williams, 2020^24^	United Kingdom	123		0 mm
	Bolas-Colvee et al, 2018^25^	Spain	250		0 mm
	Cracel-Noguiera & Pinho, 2013^26^	Portugal	634		<1 mm
	Espana et al, 2014^27^	Spain	192		0 mm
	Kerosuo et al, 1995^28^	Finland	1007	Broad median diastema (4 mm) makes someone appear less intelligent, beautiful, and socially attractive and in a lower social class	
	Noureddine et al, 2014^29^	France	105		0 mm
**America**	Kokich et al, 2006^30^	USA	203		≤2 mm
	Rosentiel & Rashid, 2002^31^	USA	1934		0 mm
	Sauders et al, 2011^32^	USA	60	The presence of a diastema is marginally better aesthetically than a midline diastema	
**South America**	Bernabae & Flores-Mir, 2007^33^	Peru	267		0 mm
	Chaves et al, 2021^34^	Brazil	102		≤0.5 mm
	Malheiros et al, 2018^35^	Brazil	130		0 mm
	Reis et al, 2020^7^	Brazil	135		0 mm
**Africa**	Athumani et al, 2006^36^	Tanzania	200		0 mm
	Aoumae, 2018^37^	Morocco	100		≤2 mm
	Houacine & Awooda, 2017^38^	Sudan	156		0 mm
**Asia**	Omar & Tai, 2014^39^	Malaysia	198		0 mm
**India**	Kumar et al, 2012^40^	India	80		≤1 mm
	Prasad et al, 2018^41^	India	150		0 mm
	Sridharan et al, 2016^42^	India	104		0 mm
	Thomas et al, 2011^43^	India	260		0 mm
**Multicentre: Middle East, Asia, Europe, and America**	Carlsson et al, 1998^44^	Sweden Canada Switzerland Japan Singapore Germany	654		0 mm
**Multicentre: America, Middle East, and Asia**	Kao et al, 2009^45^	USA Vietnam Kuwait	225	Middle Eastern individuals found a 1- to 1.5-mm diastema more aesthetic than did Europeans	

## Results

### Representation of the West African region

#### Location

All the papers that met the inclusion criteria except one^52^ were from Nigeria. Given that there are 16 countries in West Africa, the available evidence is skewed to a Nigerian perspective. Interestingly, only 3 other papers from Africa were noted during the literature search,^36^, ^37^, ^38^ revealing the deficit of research in this topic on the continent.

In all cases, the lead researchers were affiliated with dental departments in government hospitals. In Nigeria, all these universities were based in South Nigeria. The study from Ghana^52^ was based in 2 rural communities in the same district of Northern Ghana.

Based on the data available, this review speaks predominantly to opinions within the Nigerian population at best and, perhaps more selectively, the Southern coast of Nigeria.

#### Sample population

The papers represent 3553 participants, all of whom are aged 16 years or older, who identify as Black West Africans. The prevalence of MMDs in the sample ranged from 16.9%^51^ to 38.5%.^52^

In the case of 4 papers, the participants for the study were taken from the institutions where the researchers were based.^2^^,^^51^^,^^53^^,^^54^ For the remaining 4 papers, 1 was based in a private clinic,^5^ 1 was based in secondary and tertiary educational institutions,^49^ and 2 were based amongst communities in regions of their respective countries.^50^^,^^52^

Akinboboye et al^50^ used a robust sampling method, a stratified random sample, ensuring that representation of every region of Nigeria was included in their research. The results of this paper are therefore considered generalisable.

### Demographics

The demographic information available for the samples studied varied considerably (Table 5). The heterogeneity of the demographic data collected means that limited conclusions can be drawn about the impact of age, ethnic group, socioeconomic status, or dental influence (being a dentist, dental student, dental technician, or other allied oral health professional/student) on perceptions about diastemas. The data available does allow us to examine the impact of gender and the presence of one's own diastema on perceptions.

**Table 5:** Demographic information available for included studies.

Paper	Age	Sex	Ethnic group	Socioeconomics	Dental influence	Presence of diastema
Omotoso & Kadir (2009)^53^		✔				✔
Akinboboye, Umesi & Ajayi (2015)^50^	✔	✔	✔	✔		✔
Akinbobye, Umesi, & Esan (2018)^51^	✔	✔	✔		✔	✔
Oji & Obiechina (1994)^49^		✔				✔
Umanah, Omogbai, & Osagbemiro (2015)^5^		✔	✔	✔		✔
Temisanren & Opeodu (2020)^54^	✔	✔			✔	✔
Mabiaku, Ibhawah, & Ogbe, 2017^2^	✔	✔				✔
Sackeyfio et al, 2020^52^	✔	✔				✔

### Critique of methodology

The Witt and Flores-Mir^55^ methodological scoring system was used to assess each paper's quality of evidence (Table 6). The criteria were modified by the addition of a “0” score in the categories: presentation of photos, viewing protocol and scoring technique, as in some of the research, photos were not used rendering these categories “not applicable.” This meant that each paper could score a minimum of 2 points and a maximum of 22 points.

**Table 6:** Methodological scores based on the Witt and Flores-Mir^55^ criteria.

Paper	Participant number(M:F)	Sampling method	Presentation of photos	Viewing protocol	Intra-examiner reliability	Scoring technique	Methodological score
Omotoso & Kadir (2009)^53^	589 (318:271) (unknown number of laypersons)	Random sample from university students INCLUSION CRITERIA • Speaks Yoruba • Family resident in state	Self-administered questionnaire with no photos; participants commented on their own diastema	Not applicable	Not mentioned	Not applicable; binary decision on attractiveness and desirability	9/22
	**4/4**	**3/3**	**1/6**	**0/4**	**1/2**	**0/3**	
Akinboboye, Umesi, & Ajayi (2015)^50^	402 (184:218) (unknown number of laypersons)	Stratified random sample, followed by opportunistic sampling from numerous clinics across the country INCLUSION CRITERIA • At the selected institution • No pathological drift	Colour photos with diastemas of 2 to 6 mm. No mention of type of photo	Structured paper questionnaire. No mention of how patients viewed photos	Not mentioned; researcher used to collect data.	5-point Likert scale	15/22
	**4/4**	**2/3**	**4/6**	**1/4**	**1/2**	**3/3**	
Akinbobye, Umesi, & Esan (2018)^51^	89 (45:44) (47 laypersons)	Opportunistic sample at a university INCLUSION CRITERIA • Aged 19–49 • No eye defects • Had not had orthodontic treatment	African female smile excluding nose and chin. Diastemas of 2 to 6 mm	Not mentioned	Not mentioned	5-point Likert scale	15/22
	**3/4**	**2/3**	**5/6**	**1/4**	**1/2**	**3/3**	
Oji & Obiechina (1994)^49^	1868 (ratio not documented) (unknown number of laypersons)	Randomly selected from secondary and tertiary schools INCLUSION CRITERIA • Nigerian • Aged 16+ years	Participants did not view photos	Not applicable	Not mentioned	Not applicable; binary decision on aesthetics	9/22
	**4/4**	**3/3**	**0/6**	**0/4**	**1/2**	**0/3**	
Umanah, Omogbai, & Osagbemiro (2015)^5^	141 (58:83) (unknown number of laypersons)	Opportunistic: patients from one private clinic INCLUSION CRITERIA • Attended clinic in time frame	Participants did not view photos	Not mentioned	Not mentioned	Not applicable; binary decision on attractiveness and desirability	7/22
	**4/4**	**2/3**	**1/6**	**0/4**	**1/2**	**0/3**	
Temisanren & Opeodu (2020)^54^	164 (16:148) (164 laypersons)	Opportunistic sample from a class in university INCLUSION CRITERIA • Attending clinical course at the hospital • Not had orthodontic treatment	Participants rated their own smile	Not applicable	Not mentioned; researcher used to collect data.	Not applicable; binary decision on attractiveness and desirability	8/22
	**4/4**	**2/3**	**1/6**	**0/4**	**1/2**	**0/3**	
Mabiaku, Ibhawah, & Ogbe, 2017^2^	100 (16:84) (100 laypersons)	Opportunistic sample of students at a teaching hospital INCLUSION CRITERIA • Student at the hospital	Participants rated their own smile	Not applicable	Not mentioned	Not applicable; binary decision on attractiveness and desirability	8/22
	**4/4**	**2/3**	**1/6**	**1/4**	**1/2**	**0/3**	
Sackeyfio et al., 2020^52^	200 (86:114) (200 laypersons)	Opportunistic sample from randomly selected community INCLUSION CRITERIA • Adult resident in that community	Participants did not view photos	Not applicable	Not mentioned	Not applicable; binary decision on attractiveness and desirability	9/22
	**4/4**	**3/3**	**1/6**	**0/4**	**1/2**	**0/3**	

The Akinbobye et al papers^50^^,^^51^ provide the most robust methodology, scoring 15 out of 22, with all the other papers scoring between 7 and 9.^2^^,^^5^^,^^49^^,^^53^^,^^54^ This compares poorly to the scores represented in the original systematic review in the Witt and Flores-Mir paper,^55^ where all studies scored between 13 and 20, and a subsequent systematic review by Parrini et al,^56^ where all studies scored between 13 and 21. None of the studies in this literature review were included in either systematic review.

Whilst all the researchers used a structured questionnaire, there was some variation in the content. The evidence can be split into 2 groups. Group A papers^50^^,^^51^ looked specifically at the impact of different diastema widths using altered images, whilst Group B papers^2^^,^^5^^,^^49^^,^^52^, ^53^, ^54^ looked at general opinions about diastemas. Papers in Group B asked respondents to refer to their own diastema and/or asked participants about the desirability of a midline diastemas which had naturally occurred or had been created artificially.

### Aesthetic findings

#### Experience

**Fig. 3:**
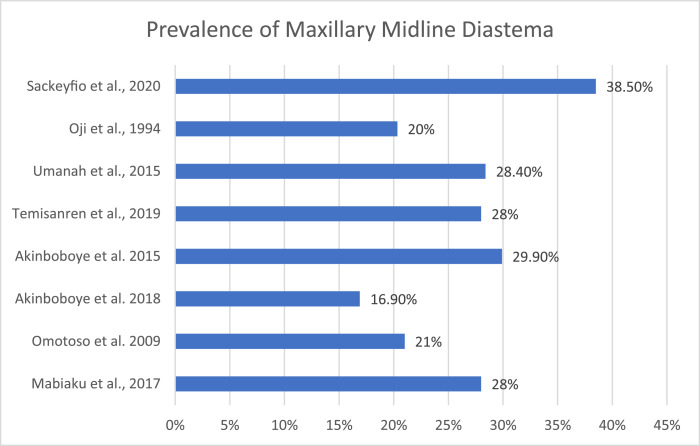
Prevalence of maxillary midline diastemas.

All 8 papers show that MMDs are a regular occurrence in this population (Figure 3). These figures are higher than the 3.5% reported in Caucasian populations.^1^

#### Beauty

**Fig. 4:**
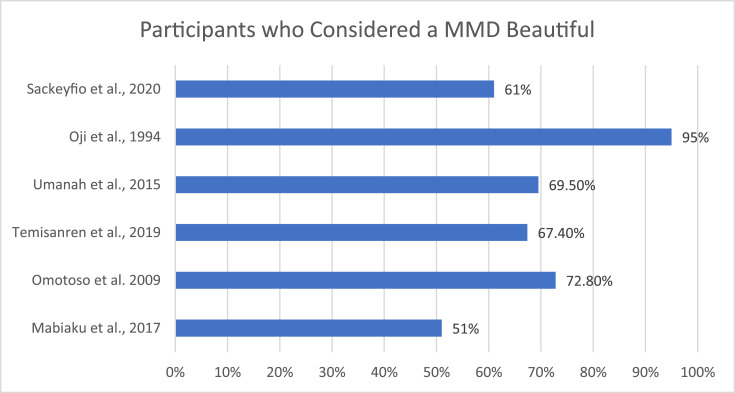
Percentage of participants who considered a maxillary midline diastema a sign of beauty.

**Fig. 5:**
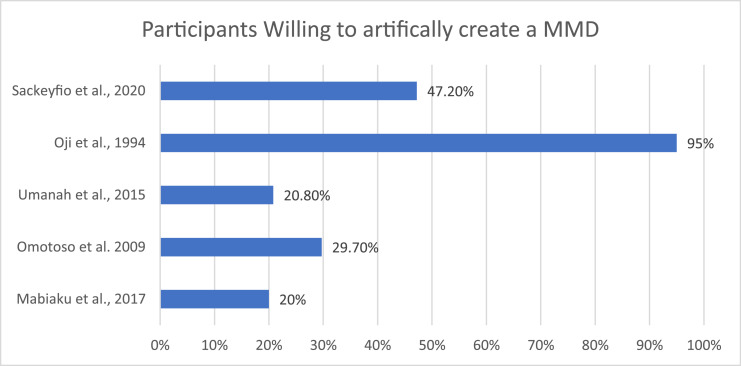
Percentage of participants willing to artificially create a maxillary midline diastema.

The evidence suggests that the MMD is considered beautiful within a Nigerian and Ghanaian population^2^^,^^53^^,^^54^ (Figure 4). Omotoso and Kadir^53^ found that 72.8% of respondents considered a midline diastema beautiful and 29.7% of their participants who did not have a natural midline diastema were interested in the artificial creation of one (Figure 5). This is compared to Sackeyfio et al^52^ in Ghana, who reported that 61% of participants found them beautiful and 47.2% of those without a diastema were willing to have one created artificially. Umanah et al^5^ reported that 44.8% of female and 19.0% of male participants who had a midline diastema had acquired it via artificial creation. It was also shown that those who had a midline diastema themselves had an increased preference for midline diastemas.^50^

### Demographic groupings

Minimal differences in opinion were noted across ethnic,^50^ socioeconomic,^50^^,^^51^ gender,^50^^,^^51^ or age groups.^51^ Those who were medically or dentally inclined liked midline diastemas less^51^ than laypersons, with the threshold for an attractive compared to an acceptable midline diastemas being 1 mm smaller for those with a medical or dental inclination.

### Preferences

A diastema of 2 to 3 mm^49^^,^^50^ or 2 to 4 mm^51^ was deemed the most attractive. Interestingly, Oji and Obiechina^49^ found that a diastema of 2 to 3 mm was the most commonly occurring amongst their participants with existing diastemas, representing 48.6% of participants, and Akinboboye found that an individual with a diastema of ≥4 mm was more likely to seek treatment.^50^ Although Sackeyfio et al^52^ did not include diastema widths in their research, they did find that 77% of participants with an MMD would not wish to have it closed. Similarly, Mabiaku et al^2^ found that 86% of their participants felt that midline diastemas should not be treated when they naturally occur in a smile.

MMDs were preferred on females,^2^ although some males considered them to be a handsome feature in men.^54^

## Discussion

### Evidence quality

Although the evidence supports an MMD as an aesthetic feature in a West African smile, the findings are not robust, lack validity (as 0 mm was not included as an option in Group A studies), and have limited representation of the West African region. It is likely that the absence of a 0-mm and 1-mm diastema in these surveys however, is due to the overall acceptability of diastemas in the region.

Despite having reasonable sample numbers, the existing data is biased. The sample population does not provide a balanced representation of the region geographically (with the exception of one of the Akinboboye studies^50^) or socioeconomically (sampling was limited to discrete areas and primarily done in an opportunistic fashion).

The information on the questionnaires themselves lacks detail. For the first Akinbobye study,^50^ for instance, we do not know the race, gender, or age of the model used in the photographs. We do not know how many photos were shown; whether these were of the same model; whether the photos were full-face, peri-oral, or retracted; and whether participants were able to view photos multiple times. Fortunately, most of these weaknesses were addressed in their subsequent study.^51^

Further research in this area should take the Witt and Flores-Mir^55^ criteria into consideration in their study design and include a more robust framework for the collection of demographic data to ensure a more robust contribution to the body of evidence.

### Global perceptions

The papers included in Table 4 reveal not only a skew in worldwide perceptions compared to those in West Africa but also a skew in the available data. This may be for a number of reasons, including lack of access to funding to generate research, lack of interest in the topic, or lack of opportunities to publish data that exists. With the majority of these papers citing a maximum of 1 mm as an acceptable diastema width, and taking into consideration where seminal textbooks are written, there is no surprise that this skew is found widely in academic literature and then translated into clinical practice.

### Shifting trends in West Africa

In West Africa, the commonly described midline diastema ideals did not hold true in pre-Colonial and Colonial times when a diastema was not perceived as a hinderance to beauty. The impact of colonialisation affected all levels of society, and there has been an instilled belief that the closer one can get to looking like “a White man,” the more successful and revered one will be. This transition can still be seen today in how clothing, hair, and diet are used as status symbols. In the same way, there has been an increased interest and availability of cosmetic dental treatments available, with some individuals being willing to travel abroad to obtain treatment. This may be in part the pursuit of a Western standard of beauty^57^ and therefore a result of cultural influence from Europe and America.^53^

Recently, there has been a shift in how women in particular are presented in the fashion and beauty industries, pivoting away from the pursuit of White standards of beauty as the accepted norm and embracing the diversity that exists in normal life.^58^ As a result, we are seeing more individuals in the media who have unique features, like an MMD, and as a result, this feature has experienced a type of renaissance recently.^59^^,^^60^

### Study limitations

The aim of this study was to explore perceptions in West Africa; however, given that papers were only identifiable from 2 West African countries, the data is limited. It is possible that there is more research into this topic in the region that remains unpublished, in addition to those papers that did not meet the inclusion criteria. Given that this study only examined peer-reviewed papers at evidence levels 1 through 3, such information could not be included. A further study with broader inclusion criteria would allow more data from the region to be captured.

Given the density of data from Nigeria, and the lack of it from elsewhere, the findings of this study are most generalisable to that specific population. Moreover, the heterogeneity of the data collected by the various authors and the variations in their study designs limited the ways in which that data could be combined and prevents further statistical analysis.

## Conclusions

The interaction between perceived attractiveness and diastema width is incredibly complex, and it is impacted by more than just diastema width. This literature review shows that MMDs are desirable and that they should be kept between 2 and 4 mm wide in this population. The practice of artificial diastema creation in some parts of Nigeria^2^^,^^5^^,^^49^^,^^53^ and Ghana^52^ and its re-creation after orthodontic treatment^4^ suggest that it should be an important consideration in the smile-design process, especially where a diastema already exists. Diastemas are frequently considered an enhancement to beauty, making them highly desirable for some individuals. It is therefore important when designing a smile for an individual from these heritages to take this into consideration.

## Conflict of interest

None disclosed.
